# Postoperative Recurrence of Medication-Related Osteonecrosis of the Jaw: A Retrospective Study of 150 Patients Undergoing Surgery

**DOI:** 10.7759/cureus.62930

**Published:** 2024-06-22

**Authors:** Shunsuke Sawada, Yuki Sakamoto, Mako Kirihigashi, Yuka Kojima

**Affiliations:** 1 Department of Dentistry and Oral Surgery, Kansai Medical University Hospital, Hirakata, JPN; 2 Department of Oral Surgery, Kansai Medical University Medical Center, Moriguchi, JPN

**Keywords:** osteosclerosis, periosteal reaction, osteolysis, recurrence, medication-related osteonecrosis

## Abstract

Introduction

Surgery is the recommended treatment for medication-related osteonecrosis of the jaw (MRONJ). However, the disease may recur postoperatively. We reviewed imaging findings in patients undergoing three or more surgeries.

Patients and methods

One hundred fifty patients with MRONJ underwent surgery at our hospital. Here, we present the characteristics of 34 surgeries in nine patients (two men and seven women; mean age, 73.9 years) who underwent surgery at least three times.

Results

Three and six patients had maxillary and mandibular lesions, respectively. The primary disease was malignancy in eight patients, and denosumab was used in seven patients. All patients initially underwent either partial maxillectomy or marginal mandibulectomy, and segmental mandibulectomy was not performed. The number of surgeries ranged from three to six (average, 3.8). Healing was eventually achieved in seven cases, but not in two cases. Of the 27 unsuccessful surgeries, postoperative cone-beam computed tomography revealed no residual osteolysis, periosteal reaction, or osteosclerosis after seven surgeries and some residual lesions after 19 surgeries; imaging was not performed after one surgery. In contrast, among the seven successful surgeries, no residual osteolysis, periosteal reaction, or osteosclerosis was observed in all six cases in which postoperative computed tomography was performed.

Conclusion

Recurrence is more common in patients with residual areas of osteolysis, periosteal reactions, or mixed-type osteosclerosis, and including these areas in the resection is desirable.

## Introduction

Medication-related osteonecrosis of the jaw (MRONJ) is an intractable form of osteonecrosis of the jaw associated with bisphosphonate (BP) and denosumab (DMB) administration for the treatment of osteoporosis and malignancy [1,2]. Previously, conservative treatments such as antimicrobial mouthwashes and oral antimicrobial agents were recommended as the first-line treatment for MRONJ [3,4]. Nonetheless, several recent studies have reported the superiority of surgical treatment [5-7]; however, no study has examined the extent of bone resection in detail. At our institution, we proactively use surgical treatment for early-stage MRONJ with good results. However, the disease often recurs after surgery, necessitating additional surgery [8]. Therefore, a detailed study on patients who experience recurrence after surgery is warranted. This case series included patients who required three or more surgeries, particularly those with postoperative residual lesions such as osteolysis, mixed-type osteosclerosis [9], and gap- or irregular-type periosteal reaction [10], on cone-beam computed tomography (CBCT) performed before and after each surgery. The study aimed to establish the optimal treatment method for MRONJ and determine the extent of bone resection during surgery.

## Materials and methods

Patients

Of 150 MRONJ patients who underwent surgery at the Department of Dental Surgery, Kansai Medical University Hospital during a seven-year period from April 2015 to March 2022, 22 patients (14.7%) developed local recurrence after surgery. Among them, nine patients (34 surgeries in total) who had postoperative recurrence and underwent three times or more surgeries were included in the study.

Factors examined

The following factors were retrospectively examined from medical records and images: age, gender, MRONJ site (maxilla/mandible), MRONJ stage [3], anesthetic method (local and general anesthesia), surgical method, and imaging findings. Surgical methods are classified as partial resection such as removal of sequestrum, marginal resection of the mandible and partial resection of the maxilla, and segmental resection such as segmental mandibulectomy and hemi-mandibulectomy. Imaging findings such as osteolysis, osteosclerosis, and periosteal reaction were determined from preoperative and postoperative CBCT for each procedure. Osteosclerosis was classified into two types: uniform-type, which shows uniform effect images, and mixed-type, which contains many small permeation images within the sclerotic image [9]. Periosteal reactions were classified into three types according to the report of Soutome et al. [10]: attached-type in which new bone is formed parallel to the mandible without the presence of gap between the mandible and new bone, gap-type in which new bone is formed parallel to the mandible with the presence of gap between the mandible and new bone, and irregular-type showing an irregularly shaped periosteal reaction. The postoperative residuals of osteolysis, osteosclerosis, and periosteal reaction were also examined by CBCT before and after each surgery. Treatment outcome was defined as cure if all clinical symptoms including bone exposure were resolved, and non-cure otherwise.

Ethics

The study protocol conformed to the ethical guidelines of the Declaration of Helsinki and the ethical guidelines for medical and health research involving human subjects by the Ministry of Health, Labor, and Welfare of Japan. Ethical approval was obtained from the Institutional Review Board (IRB) of Kansai Medical University (approval number: 2022166). The requirement for informed consent was waived because this was a retrospective study, and the research plan was published on the homepage of the hospital, highlighting the guaranteed opt-out opportunity.

## Results

Case series

**Figure 1:**
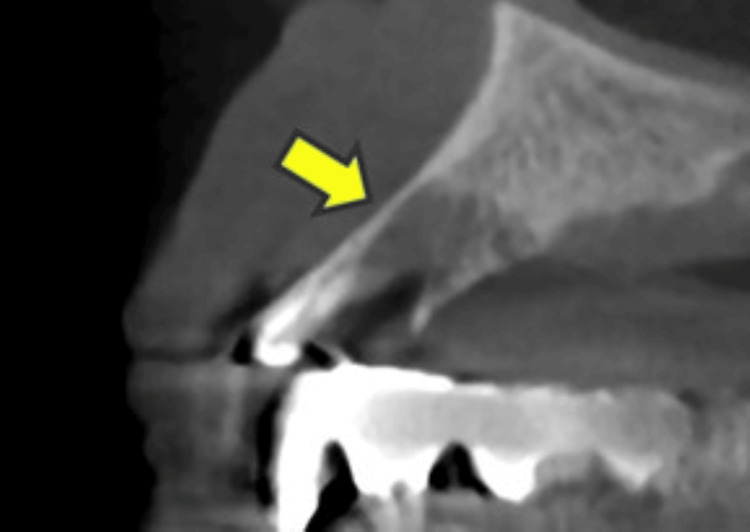
Case 1 Removal of the sequester was performed under local anesthesia. Residual osteolytic lesion (arrow) was observed, and she did not obtain cure (after the first surgery).

Case 1 involved a 76-year-old woman who was diagnosed with stage 2 maxillary MRONJ. She had been receiving BP and DMB for 53 months owing to multiple myeloma. Osteolysis that did not reach the floor of the maxillary sinus was observed. The definitive cure was achieved after three partial resections under local anesthesia and two partial resections under general anesthesia with primary closure using mucoperiosteal flaps (Figure 1).

**Figure 2:**
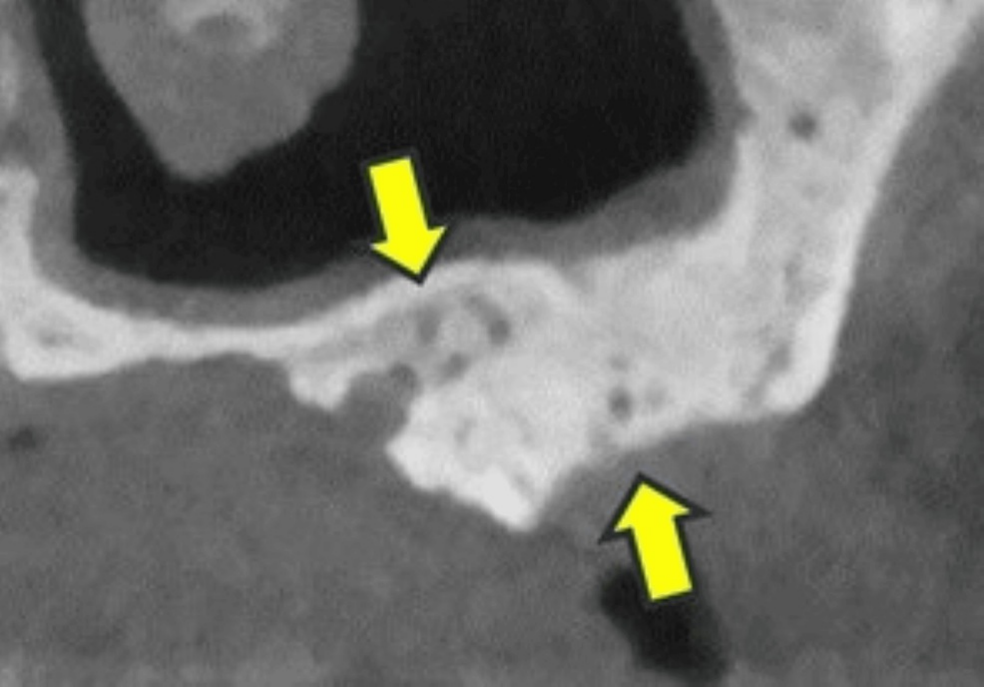
Case 2 Mixed-type osteosclerosis remained after the third surgery under local anesthesia (arrows). She finally obtained cure after the fourth surgery under general anesthesia.

Case 2 involved a 78-year-old woman who was diagnosed with stage 2 maxillary MRONJ. She had been receiving BP and DMB for 75 months for multiple myeloma. Osteolysis that did not reach the floor of the maxillary sinus and mixed-type osteosclerosis were observed. Three partial resections combined with closure using mucoperiosteal flaps were performed under local anesthesia (Figure 2); however, bone exposure recurred. Therefore, subsequent surgery was performed under general anesthesia, resulting in healing.

**Figure 3:**
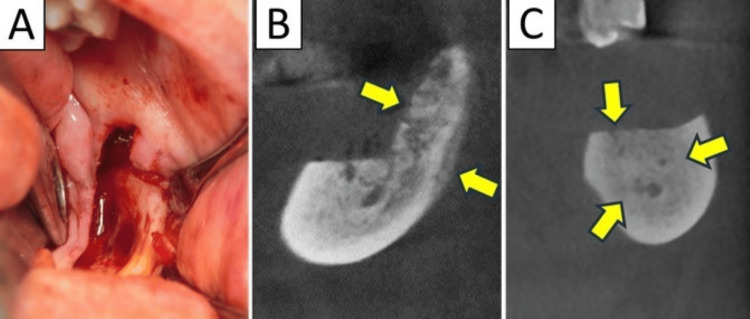
Case 3 In the first surgery, intraoperative findings showed that the bone that appeared to be necrotic bone was removed (A), but the osteolysis and periosteal reaction remained (arrows) and did not heal (B). The third surgery was performed to remove the osteolysis and periosteal reaction areas, but healing was not achieved. Postoperative imaging (C) showed mixed osteosclerosis (arrows).

Case 3 involved a 78-year-old woman who was diagnosed with stage 2 mandibular MRONJ. She had been receiving DMB for 30 months for multiple myeloma. CBCT showed osteolysis, gap-type periosteal reaction and mixed-type osteosclerosis. Marginal mandibulectomy up to the region where bleeding was observed intraoperatively and closure using a mucoperiosteal flap were performed three times under general anesthesia; however, no cure was achieved. Two additional partial resections were performed, but no cure was achieved (Figures 3A-3C).

**Figure 4:**
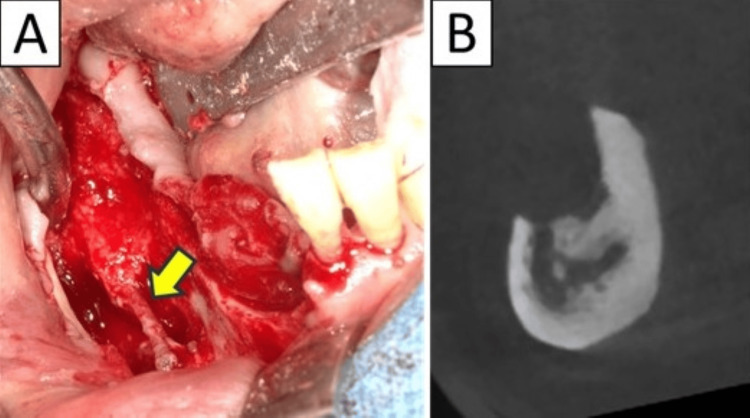
Case 4 The inferior alveolar neurovascular bundle (arrow) was detected and preserved (A), while the surrounding osteolytic area was adequately curetted (B), and good result was obtained (the third surgery).

Case 4 involved a 74-year-old man with stage 2 mandibular MRONJ. He had been receiving low doses of BP for at least five years to manage cancer treatment‑induced bone loss (CTIBL). CBCT revealed osteolysis involving the mandibular canal and mixed-type osteosclerosis. The patient underwent three mandibulectomies with primary closure using mucoperiosteal flaps under general anesthesia. In the third surgery, adequate curettage of the osteolytic area was performed while preserving the inferior alveolar neurovascular bundle (Figures 4A, 4B), and cure was achieved.

**Figure 5:**
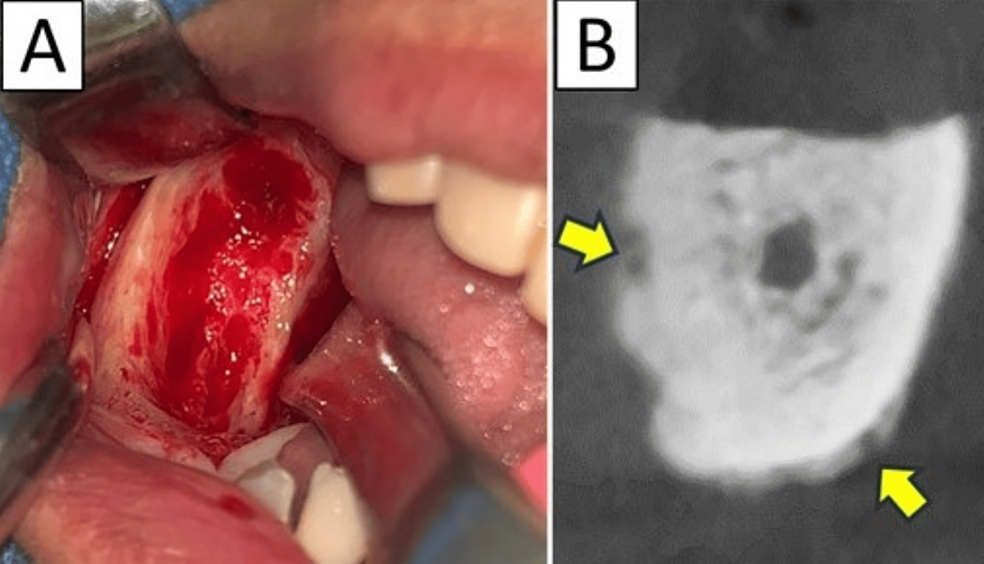
Case 5 Marginal mandibulectomy was performed twice, but no cure was obtained (A). After the second surgery, periosteal reaction (arrows) appeared (B), and finally she underwent segmental mandibulectomy.

Case 5 involved a 72-year-old woman with stage 2 mandibular MRONJ. She had been receiving denosumab for 24 months for bone metastasis from breast cancer. Initially, CBCT showed no osteolysis; however, mixed-type osteosclerosis was observed. Marginal mandibulectomy and closure using a mucoperiosteal flap were performed under general anesthesia; however, no cure was achieved. Subsequently, osteolysis was observed around the resection site. Marginal mandibulectomy and closure using a mucoperiosteal flap were performed again under general anesthesia; however, no cure was achieved. After the second surgery, irregular-type periosteal reaction appeared on CBCT (Figures 5A, 5B). Subsequently, segmental mandibulectomy was performed, which resulted in a cure.

**Figure 6:**
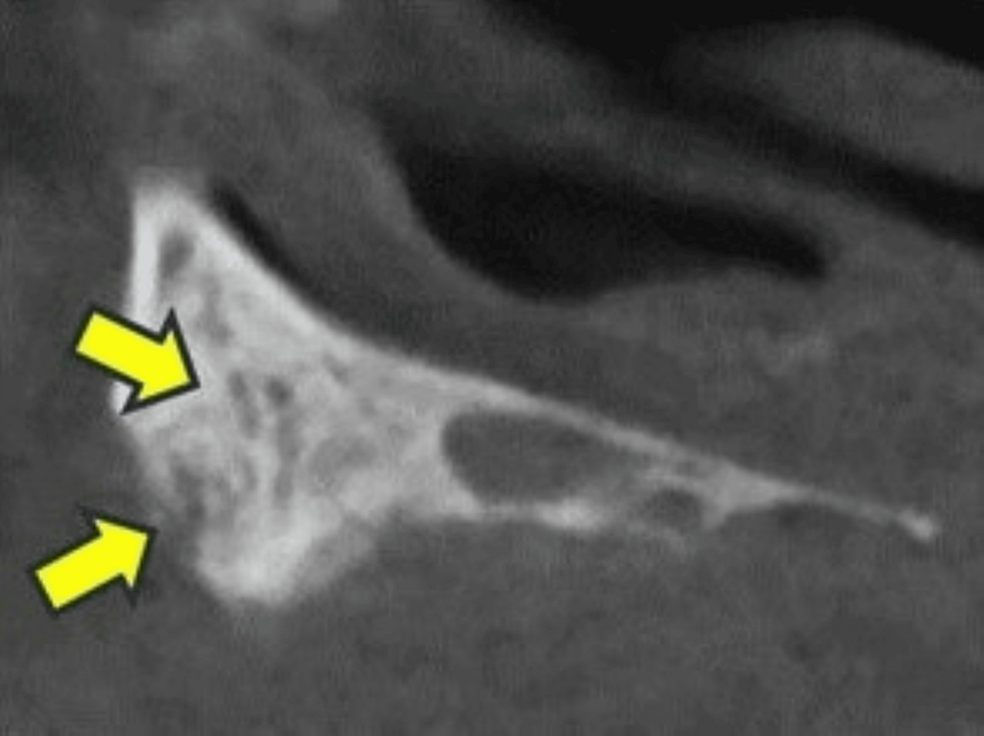
Case 6 The patient underwent three partial maxillectomies with no cure. Residual mixed-type osteosclerosis (arrows) was seen (after the second surgery).

Case 6 involved a 73-year-old woman with stage 2 maxillary MRONJ. She had been receiving denosumab for 28 months for bone metastases from breast cancer. Osteolysis that did not extend to the floor of the maxillary sinus, gap-type periosteal reaction, and mixed-type osteosclerosis were observed on CBCT. Partial maxillectomy and closure using a mucoperiosteal flap were performed under general anesthesia. The patient subsequently experienced relapse and underwent two additional resections under local anesthesia (Fig. 6); however, no cure was achieved.

**Figure 7:**
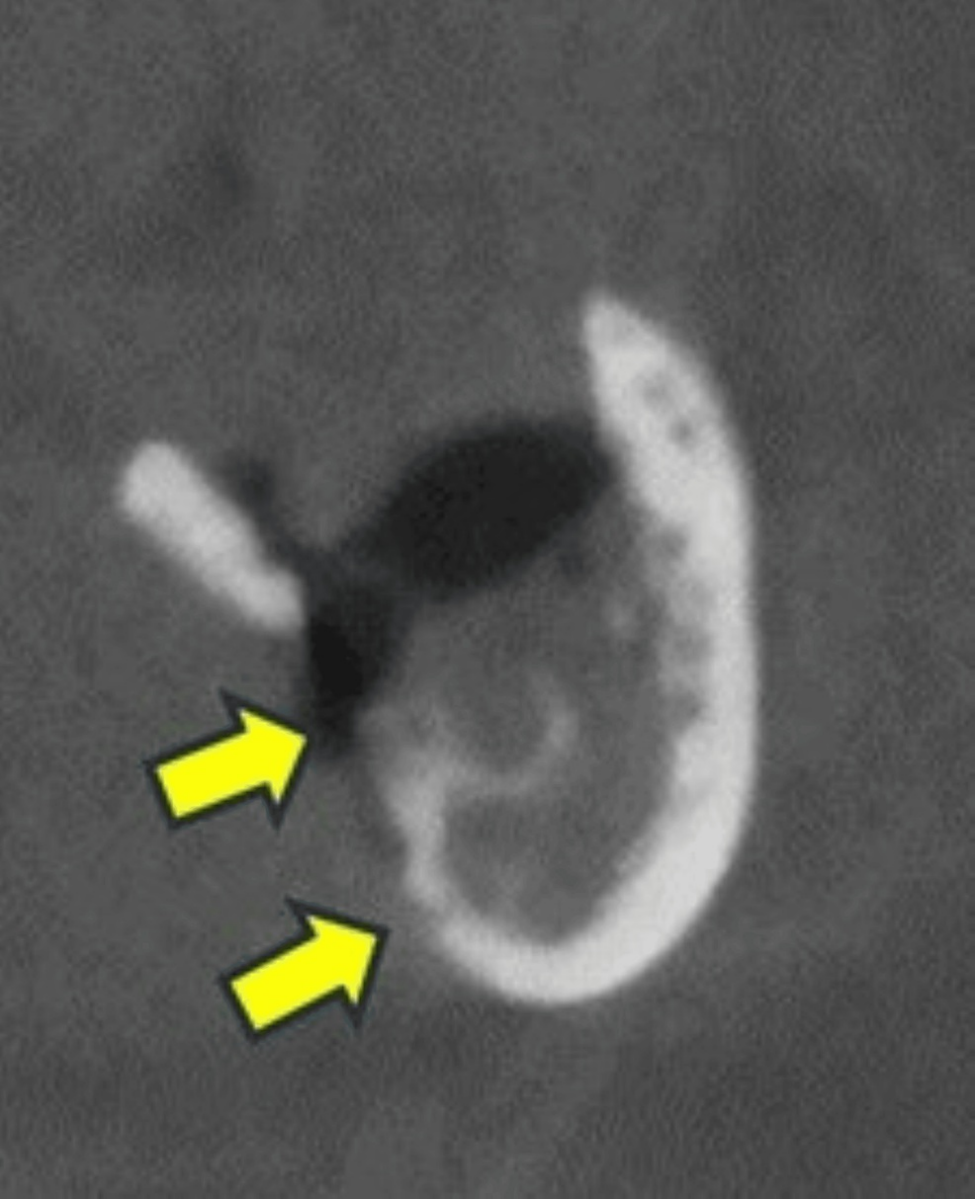
Case 7 Marginal mandibulectomy was performed, but no cure was obtained and further osteolysis in the cortical bone appeared (arrows). He ultimately underwent hemi-mandibulectomy, which resulted in healing.

Case 7 involved a 59-year-old man with stage 1 mandibular MRONJ. He had been receiving denosumab for seven months for bone metastasis from lung cancer. Initially, no signs of osteolysis were observed; however, mixed-type osteosclerosis was observed. The patient underwent a marginal mandibulectomy of the exposed bone and closure using a mucoperiosteal flap under general anesthesia. However, bone exposure and osteolysis around the resection site recurred (Figure 7). Therefore, segmental mandibulectomy was performed, but the disease recurred near the preserved mandibular condyle; ultimately, hemi-mandibulectomy was performed, and healing was achieved.

**Figure 8:**
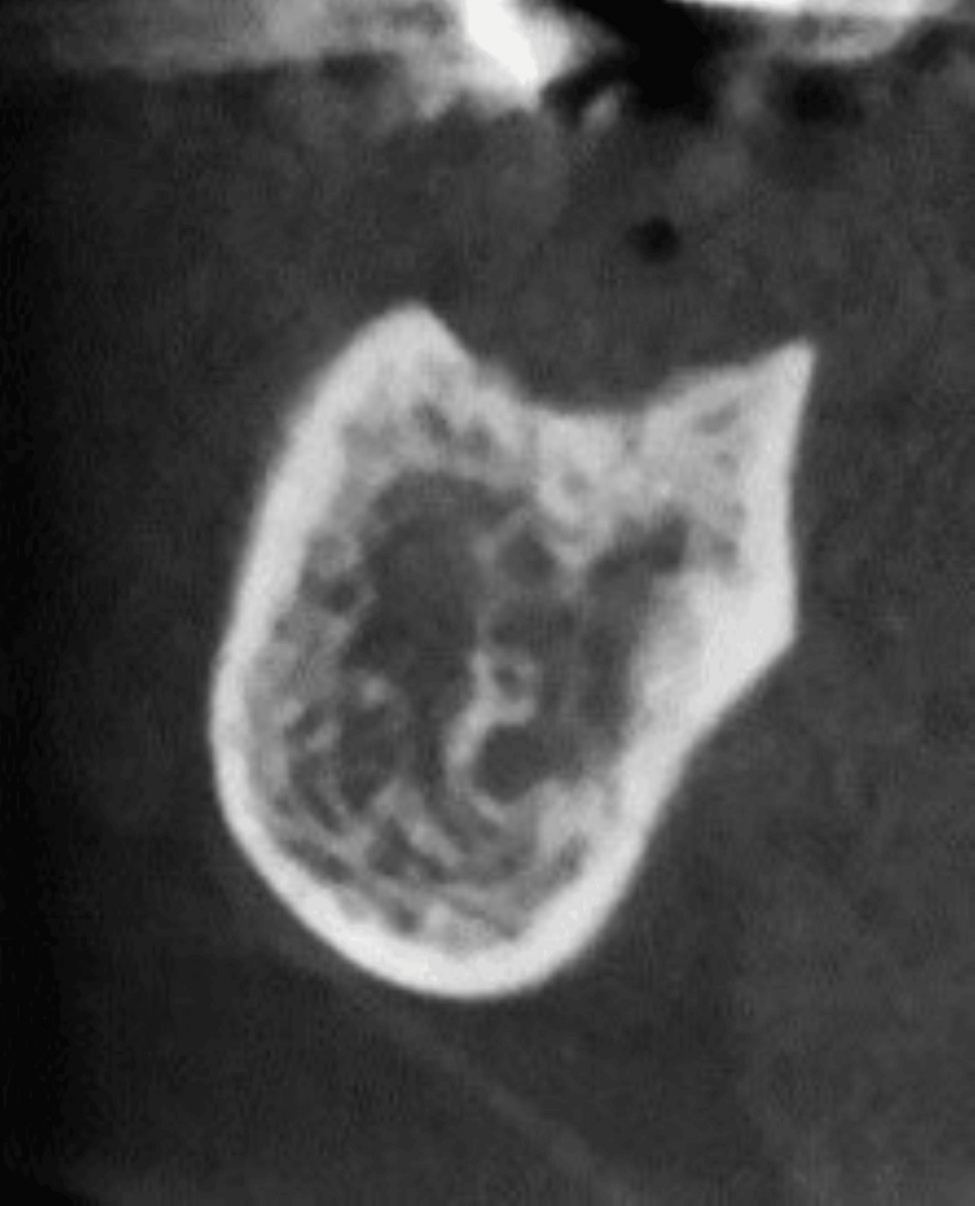
Case 8 There were no abnormal findings on CT images after the second surgery. However, four marginal resections were required before healing was achieved.

Case 8 involved a 78-year-old woman with stage 2 mandibular MRONJ. The primary disease was breast cancer, and denosumab was administered for eight months. Only panoramic radiographs were obtained before and after the initial surgery, and details of the imaging findings were unknown. Healing was achieved after marginal mandibulectomy and closure using a mucoperiosteal flap was performed twice under local anesthesia and twice under general anesthesia (Figure 8).

**Figure 9:**
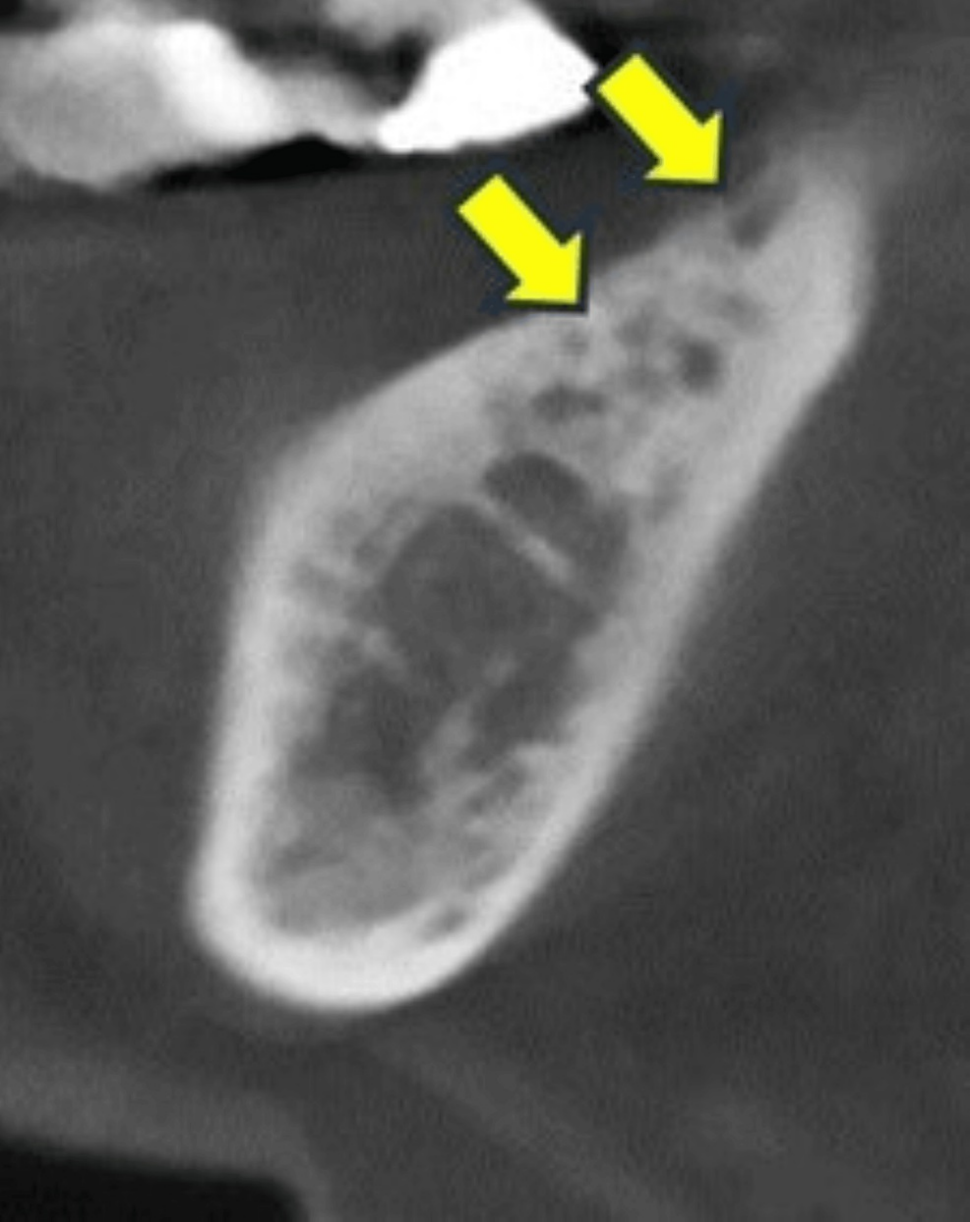
Case 9 Image after the fourth marginal mandibulectomy. Mixed osteosclerosis (arrows) was observed, resulting in recurrence. Ultimately, healing was achieved after two segmental mandibulectomies.

Case 9 involved a 79-year-old woman with stage 2 mandibular MRONJ. She had been receiving denosumab for 16 months for bone metastases from breast cancer. CBCT showed no osteolysis, periosteal reaction, or mixed-type osteosclerosis. Marginal mandibulectomy with mucoperiosteal flap closure was performed thrice under local anesthesia and once under general anesthesia (Figure 9), but cure was not obtained. She underwent two segmental mandibulectomies under general anesthesia, and after the fifth surgery, healing was achieved.

Pre- and postoperative CBCT findings

Pre- and postoperative CBCT findings are summarized in Table 1. Of the 27 surgeries in which no cure was achieved; no residual osteolysis, periosteal reaction, or osteosclerosis on postoperative CBCT was observed after seven surgeries; some residual lesions were observed after 19 surgeries; and postoperative imaging studies were unavailable for one surgery. In contrast, of the seven surgeries in which healing was achieved, no residual osteolysis, periosteal reaction, or osteosclerosis was observed in all six cases for which postoperative CBCT was obtained.

**Table 1:** Pre- and postoperative CBCT findings and treatment outcome partial: removal of sequestrum, partial maxillectomy and marginal mandibulectomy; segmental: segmental mandibulectomy or hemi-mandibulectomy; n.e.: not examined; CBCT: cone-beam computed tomography.

No.	Anesthesia	Operation method	Preoperative CT findings			Residuals on postoperative CT images			Treatment outcome
			Osteolysis	Periosteal reaction	Mixed-type osteosclerosis	Osteolysis	Periosteal reaction	Mixed-type osteosclerosis	
Case 1-1	local	partial	localized	-	+	localized	-	+	non-healing
2	local	partial	localized	-	+	-	-	+	non-healing
3	local	partial	localized	-	+	-	-	+	non-healing
4	general	partial	localized	-	+	localized	-	+	non-healing
5	general	partial	localized	-	+	-	-	-	healing
Case 2-1	local	partial	localized	-	+	-	-	+	non-healing
2	local	partial	localized	-	+	localized	-	+	non-healing
3	local	partial	extended	attached-type	+	extended	-	-	non-healing
4	general	partial	n.e.	n.e.	n.e.	n.e.	n.e.	n.e.	healing
Case 3-1	general	partial	localized	gap-type	+	+	gap-type	+	non-healing
2	general	partial	localized	gap-type	+	-	gap-type	+	non-healing
3	general	partial	localized	-	+	-	-	+	non-healing
Case 4-1	general	partial	extended	-	+	extended	-	+	non-healing
2	general	partial	extended	-	+	extended	-	+	non-healing
3	general	partial	extended	-	+	-	-	-	healing
Case 5-1	general	partial	-	-	+	-	-	+	non-healing
2	general	partial	localized	-	+	-	-	+	non-healing
3	general	segmental	-	irregular-type	+	-	-	-	healing
Case 6-1	general	partial	localized	gap-type	+	localized	gap-type	+	non-healing
2	local	partial	extended	irregular-type	+	extended	gap-type	+	non-healing
3	local	partial	extended	-	+	extended	-	+	non-healing
Case 7-1	general	partial	-	-	+	-	-	+	non-healing
2	general	segmental	extended	-	+	-	-	-	non-healing
3	general	segmental	-	-	-	-	-	-	healing
Case 8-1	local	partial	n.e.	n.e.	n.e.	n.e.	n.e.	n.e.	non-healing
2	local	partial	localized	-	-	-	-	-	non-healing
3	general	partial	-	-	-	-	-	-	non-healing
4	general	partial	localized	-	-	-	-	-	healing
Case 9-1	local	partial	-	-	-	-	-	-	non-healing
2	local	partial	-	-	-	-	-	-	non-healing
3	local	partial	-	-	-	-	-	-	non-healing
4	general	partial	-	-	+	-	-	+	non-healing
5	general	segmental	-	-	+	-	-	-	non-healing
6	general	segmental	-	irregular-type	-	-	-	-	healing

## Discussion

Previous studies [5-7] have shown that the prognosis of MRONJ is better after extensive surgery that includes the surrounding healthy bone than after conservative surgery in which only the necrotic bone is resected. In our previous multicenter study [5], the healing rates of patients who underwent conservative and extensive surgery were 17/38 (44.7%) and 106/134 (79.1%), respectively. However, recurrence is sometimes observed despite extensive surgery.

We previously reported that patients with periosteal reactions have poorer outcomes after surgery [11]. Moreover, sites with gap- or irregular-type periosteal reactions are infectious lesions and should be included in the resection [10,12]. Currently, we resect not only areas of osteolysis and sequestrum separation but also areas of periosteal reaction as much as possible during MRONJ surgery. However, this study revealed that some patients repeatedly experience recurrence and reoperation.

Osteosclerosis is a common imaging finding in patients with MRONJ. However, careful observation often shows marked bone sclerosis near the MRONJ lesion compared with the healthy side. This suggests that some bone sclerosis is caused by antiresorptive agents, while some is caused by MRONJ. Recently, Suyama et al. [9] reported that osteosclerosis around MRONJ lesions contain several small radiographic features instead of the usual uniform osteosclerosis and named it mixed-type osteosclerosis. They recommended that bones showing mixed-type osteosclerosis should be included in the resection field. In this retrospective study, mixed-type osteosclerosis was present before surgery in seven out of nine cases of recurrence, and recurrence was observed in most surgeries that did not include the sclerotic area in the resection. Further studies are required to clarify the diagnostic criteria, imaging findings, and histopathology of mixed osteosclerosis.

Currently, the extent of bone resection in MRONJ surgery is mainly determined based on the osteolysis and periosteal reaction areas on CBCT. However, in recent years, the frequency of nonosteolytic MRONJ, which does not show abnormal findings such as osteolysis or periosteal reactions on CBCT, has increased [13,14]. Case 7 was characterized by a non-osteolytic MRONJ, and although the extent of bone resection was determined based on intraoperative findings at the time of the initial surgery, the lesion could not be completely removed, resulting in enlargement that ultimately required hemi-mandibulectomy. Otsuru et al. reported poor outcomes in six cases of MRONJ with extensive periosteal reactions with no or minimal bone exposure or osteolysis [15]. For atypical MRONJ, such as nonosteolytic MRONJ and periosteal reaction-dominant MRONJ, determining the extent of bone resection based on CBCT findings is difficult, and preoperative diagnosis using other methods, such as magnetic resonance imaging or single photon emission computed tomography/CT, may be necessary in the future. This study had several limitations. Since this case series included a small number of cases, the generalizability of the results obtained remains unclear.

## Conclusions

We consider surgery to be the first-line treatment for patients with MRONJ, with good results in many cases, but recurrence may occur after surgery. In this study, we investigated the characteristics of nine patients who had recurrence after MRONJ surgery and underwent three or more surgeries. Non-healing cases were more common in those showing no osteolysis, mixed-type osteosclerosis, and periosteal reaction. In examining each surgery, postoperative CBCT showed more cases with residual areas of osteolysis, periosteal reaction, and mixed-type osteosclerosis, suggesting that the areas showing these findings should be included in the resection area.
